# Differences in Tumor Immune Microenvironment in Metastatic Sites of Breast Cancer

**DOI:** 10.3389/fonc.2021.649004

**Published:** 2021-03-18

**Authors:** Hyunjong Lee, Kwon Joong Na, Hongyoon Choi

**Affiliations:** ^1^Department of Nuclear Medicine, Seoul National University College of Medicine, Seoul, South Korea; ^2^Department of Molecular Medicine and Biopharmaceutical Sciences, Graduate School of Convergence Science and Technology, Seoul National University, Seoul, South Korea; ^3^Department of Thoracic and Cardiovascular Surgery, Seoul National University Hospital, Seoul, South Korea

**Keywords:** cancer, metastasis, tumor immune microenvironment, macrophage, neutrophil

## Abstract

**Introduction:** Tumor immune microenvironment (TIME) promotes immune escape, allowing for tumor progression and metastasis. In spite of the current evidence of the complicated role of immune cells in promoting or suppressing cancer progression, the heterogeneity of TIME according to the tumor site has been scarcely investigated. Here, we analyzed transcriptomic profiles of metastatic breast cancer to understand how TIME varies according to tumor sites.

**Methods:** Two gene expression datasets from metastatic breast cancer of various sites and a single-cell RNA sequencing dataset of primary breast cancer and metastatic lymph nodes were analyzed. The immune cell-type enrichment of each tumor was estimated. Immune cell types were identified by clustering analysis, and the proportions of cell types in TIME were assessed according to the tumor site.

**Results:** Metastatic bone lesions showed more neutrophils than breast lesions. Tumors clustered according to immune cell type were significantly associated with tumor site. In single-cell analyses, the TIMEs of metastatic lymph nodes showed fewer macrophages than those of primary tumors. Differentially expressed gene signatures in the primary tumor and metastatic lymph nodes were associated with macrophage activation.

**Conclusion:** We conclude that metastatic sites show variable enrichment patterns of immune cells, and that the TIME of metastatic lesions should be considered in precise immuno-oncology treatments.

## Introduction

The tumor immune microenvironment (TIME) plays a key role in tumor progression and metastasis. Interactions between immune cells, stromal cells, and chemokines establish a pre-metastatic niche for circulating tumor cells (1–3). The cell types in TIME play different pro- and antitumor roles during tumor progression (4). For instance, neutrophils are recruited to the metastatic site to initiate metastasis by activating dormant tumor cells and enhancing migration of tumor cells (5, 6). Tumor-associated macrophages (TAMs), a specific subtype of macrophages, facilitate tumor progression to support invasion of tumor cells and suppress the tumoricidal immune response (7–9). In contrast, CD8+ T-cells and natural killer cells inhibit tumor growth by promoting antitumor immunity (10, 11).

The roles of immune regulation in different metastatic sites have recently been elucidated (12). A metastatic niche is established in the lymph nodes by suppressing autoreactive immunity mediated by cytokines such as IL-17 and regulatory T-cells that support immune evasion of tumor cells (13, 14). In the brain, one of the most common organs of hematogenous metastasis, metastasis is associated with different cellular populations such as astrocytes, microglia, and macrophages, which play a role in cancer cell infiltration and survival (15, 16). Protecting cancer stem cells from immune cells is an important mechanism promoting metastasis in the bone (17). Despite recent findings of specific cellular populations in TIME and the close interaction between cancer cells and immune cells, to the best of our knowledge, there has been no explorative interrogation of cell enrichment within TIME according to the metastatic site. Notably, as the cellular composition of TIME can vary across tumor types and is regarded as a key predictive biomarker for immunotherapy (18, 19), a comprehensive understanding of TIME according to the metastatic site has a clinical implication with regard to tumor-specific immunotherapy (20).

Therefore, this study aimed to assess the difference in the cellular composition of TIME in different metastatic sites. We used multiple datasets, including microarray and single-cell RNA-sequencing (scRNA-seq), from breast cancer patients to assess enrichment and proportions of immune cells based on the metastatic site. In addition, the proportions of cell types in TIME were assessed in the scRNA-seq dataset. Differential gene expression analysis was conducted to compare primary breast tumors and metastatic lymph nodes.

## Methods

### Data Sources

We used four publicly available datasets in this study. For bulk tissue analyses, we obtained two microarray datasets of metastatic breast cancer tissues (accession number GSE124647 and GSE56493) from the Gene Expression Omnibus database (https://www.ncbi.nlm.nih.gov/geo/) (21, 22). The normalized gene expression data and the clinical and demographic characteristics of both cohorts were downloaded using the “GEOquery” package in R (23). Subjects with metastatic lesions in “lung/pleura” lesions were excluded due to uncertainty whether metastatic lesions located in a lung or pleura. Subjects with metastatic lesions in “others” were excluded due to a lack of data for a specific location. Subjects with ovary or soft tissue were excluded, because they were included in only one dataset (GSE124647), not in another dataset (GSE56493). A total of 102 and 117 subjects were included for the analysis in GSE124647 and GSE56493, respectively. The specific sites of breast cancer metastasis are summarized in Supplementary Table 1. For single-cell analysis, we obtained an scRNA-seq dataset of breast cancer patients from the Gene Expression Omnibus database (accession number GSE75688) (24). It included 11 breast cancer patients. Additionally, an scRNA-seq dataset of head and neck cancer (accession number GSE103322) was analyzed to validate the results of the breast cancer dataset (25). It included 17 head and neck cancer patients.

### Immune Cell Enrichment Analysis

To evaluate the cellular landscape of TIME, cell type enrichment scores and proportions of cellular populations were estimated. We used three available bioinformatics analytical tools that provide an estimation of the cell type enrichment scores or relative levels of distinct cell types from gene expression data: xCell, CIBERSORT, and TIMER (26–28). We estimated the cell type enrichment scores of 64 immune and stromal cell types of tumor microenvironment using “xCellAnalysis” function included in the “xCell” package in R. Subsequently, composite scores of 35 immune cells were selected for the analysis. Using CIBERSORT, we obtained the relative proportion of 22 immune cell types in TIME. The CIBERSORT analysis process was performed with web-based resource (https://cibersort.stanford.edu/). TIMER provided the enrichment score reflecting the relative abundance of 6 immune cell types. The TIMER analysis process was performed with web-based resource (http://timer.cistrome.org/).

### scRNA-Seq Analysis

A read count matrix was generated from an scRNA-seq dataset of breast cancer patients (GSE75688). The scRNA-seq data were scaled by log-normalization after the read counts were divided by the total number of transcripts and multiplied by 10,000. Two thousand highly variable genes were selected using the “FindVariableFeatures” function of Seurat (version 3.0) (29, 30). Data were then scaled to z-scores with regression analysis from total cellular read counts and mitochondrial read counts. Cell types were determined using the graph-based clustering approach implemented by the “FindClusters” function. Before clustering, dimension reduction was performed by principal component analysis, and 10 dimensions were used for the clustering. The conservative resolution was set to 1.0. To identify the marker genes of the clusters, the “FindAllMarkers” function of Seurat was used, and 10 high-ranked marker genes were identified according to the fold-change. The scRNA-seq data were embedded by two-dimensional projection using t-distributed stochastic neighborhood embedding. To identify cell types, the expression levels of 8 markers (*CD68, CD79A, CD3D, CD8A, CD4, FOXP3, COL1A1*, and *CDH1*) were assessed. CD3D, CD8A, CD4, and FOXP3 are well-known markers for tumor-infiltrating lymphocytes (31). CD68 and CD79A are the most representative markers of macrophages and B-cells, respectively (32, 33). CDH1 and COL1A1 are markers for epithelium and stroma (34, 35). Based on the expression levels, each cluster was classified into 4 cell types: cancer cell, T-cell, B-cell, and macrophage. Cell numbers were compared between the primary tumor and metastatic lymph nodes in the entire dataset and in the paired samples. Subsequently, differentially expressed genes (DEGs) were extracted by Wilcoxon rank sum test using the “FindMarkers” function. The threshold of adjusted *p*-value was 0.05 to determine DEGs. Genes with adjusted *p*-value below 0.1 were employed in gene ontology (GO) analysis. It was performed to assess biological processes related to the DEGs with the enrichGO function. The cutoff value for *p*-value and *q*-value was 0.05.

### Statistics

The cell type enrichment scores estimated from the primary tumor and other metastatic sites were compared using the Wilcoxon rank test. Log_2_ fold change (log_2_FC) was calculated from mean of enrichment scores of each cell type. A threshold of log_2_FC was 0.5 to determine differential expression, and a *p*-value of 0.05 was considered significant in volcano plots showing differences in cell type enrichment scores or relative levels between the metastatic site and breast lesion. Hierarchical clustering was performed in Euclidean distances between each sample with Ward's minimum variance method. Chi-square analyses were applied to identify association between clusters and metastatic sites. All statistical analyses were performed using R (v 3.6.1). The overall scheme of analysis methods is described in Supplementary Figure 1.

## Results

### Differences in Cell Types in TIME as Per the Tumor Site

**Figure 1:**
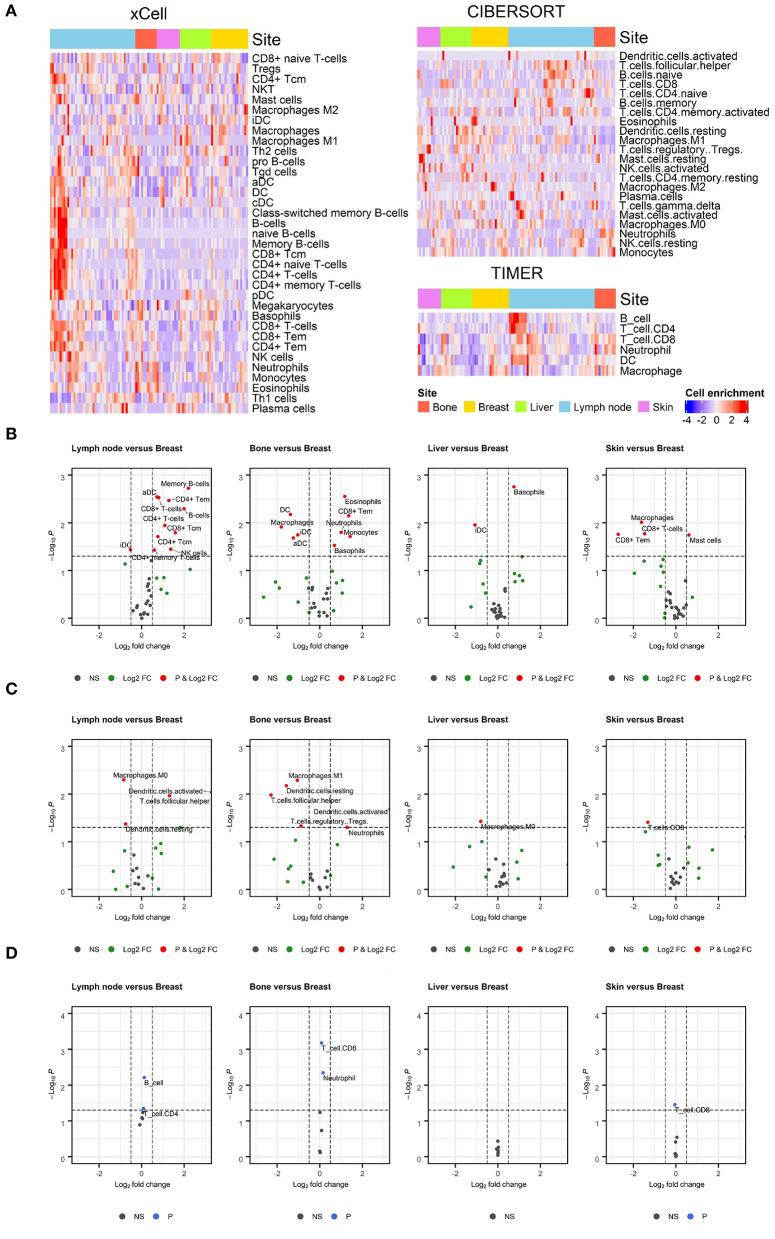
Enrichment of immune cell populations in different metastatic sites. **(A)** Heatmaps depicting the distribution of immune cell enrichment scores according to metastatic site estimated using xCell, CIBERSORT, and TIMER. The distribution of immune cells varied across all samples. **(B–D)** Volcano plots showing enriched immune cells in specific metastatic sites compared to breast lesions. Wilcoxon rank test was applied to compare the enrichment of immune cells. A threshold of log_2_FC was 0.5 to determine differential expression, and a *p*-value of 0.05 was considered significant. Values outside the range of x-axis were displayed at the margin of plots. Results from the xCell, CIBERSORT, and TIMER analyses are shown in **(B–D)**, respectively. In CIBERSORT analysis, there were fewer macrophages in metastatic lymph nodes than in breast lesions. In xCell and CIBERSORT analyses, there were more neutrophils in metastatic bone lesions than in breast lesions.

We first evaluated the enrichment of cell types in TIME using a gene expression dataset from breast cancer patients (GSE124647). As shown in the heatmap of the cell enrichment analysis, immune cells were highly different in different samples (Figure 1A). We also evaluated the effect of metastatic tumor sites on the cell enrichment scores/proportions. To identify specific cell types enriched in metastatic tumors, cell enrichment scores/proportions of each immune cell from each metastatic site were compared with those from breast lesions (Figures 1B–D). In CIBERSORT analysis, fewer macrophages were observed in metastatic lymph nodes than in breast lesions. In xCell and CIBERSORT analysis, more neutrophils were found in metastatic bone lesions than in breast lesions. Log_2_FC and *p*-value were listed in Supplementary Table 2.

### Different Myeloid Cell Compositions as Per the Tumor Site

**Figure 2:**
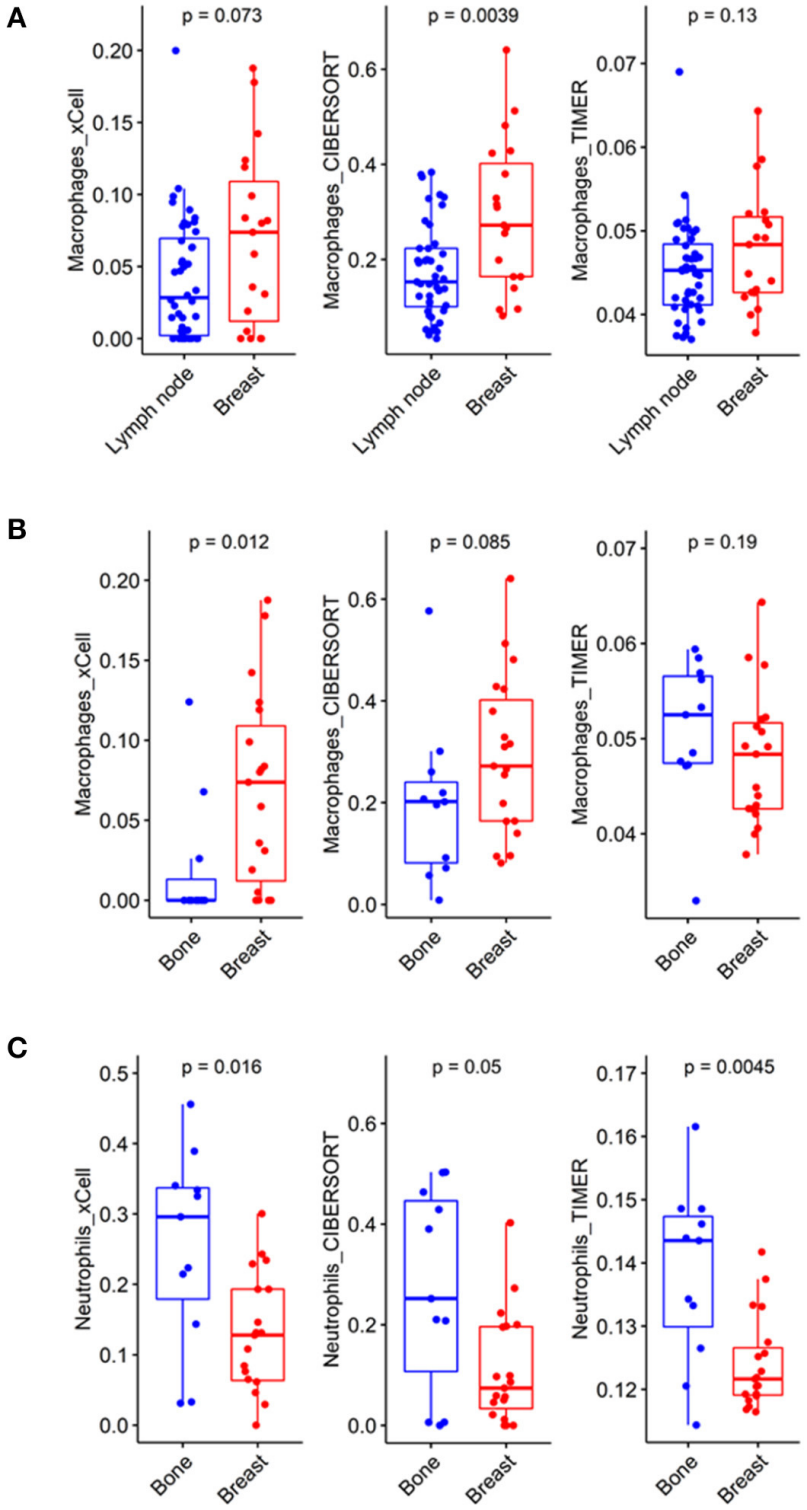
Different compositions of myeloid cells according to metastatic tumor site. **(A)** In the CIBERSORT analysis, macrophages showed significantly lower enrichment scores in metastatic lymph nodes than in breast lesions (*p* = 0.0039, CI = 0.04~0.19). **(B)** In the xCell analysis, macrophages showed significantly lower enrichment scores in metastatic bone lesions than in breast lesions (*p* = 0.012, CI = −8.38*10^−2^~-6.06*10^−5^). **(C)** In the xCell and TIMER analyses, neutrophils showed significantly higher enrichment scores in metastatic bone lesions than in breast lesions (*p* = 0.016, CI = 0.03~0.23 and *p* = 0.0045, CI = 4.83*10^−3^~0.02, respectively).

The previous analysis showed that macrophages were less enriched in some metastatic tumors compared to the primary site, especially in xCell and CIBERSORT analyses. Therefore, we focused on the enrichment level of macrophages in metastatic lymph nodes and bone lesions. Metastatic lymph nodes had significantly fewer macrophages than breast lesions in the CIBERSORT analysis (*p* = 0.004, W = 608). There was no significant difference in xCell and TIMER analyses (Figure 2A). A lower macrophage enrichment score was also observed in metastatic bone lesions. Metastatic bone lesions had fewer macrophages than breast lesions (Figure 2B). The xCell analysis showed a significantly lower macrophage enrichment score in metastatic bone lesions than in breast tumors (*p* = 0.012, W = 46). There was no significant difference in CIBERSORT and TIMER analyses. Conversely, there were more neutrophils, another myeloid cell type, in metastatic bone lesions than in breast lesions in all 3 analyses (Figure 2C, *p* = 0.016, W = 161 for xCell; *p* = 0.050, W = 150.5 for CIBERSORT; and *p* = 0.005, W = 169 for TIMER).

**Figure 3:**
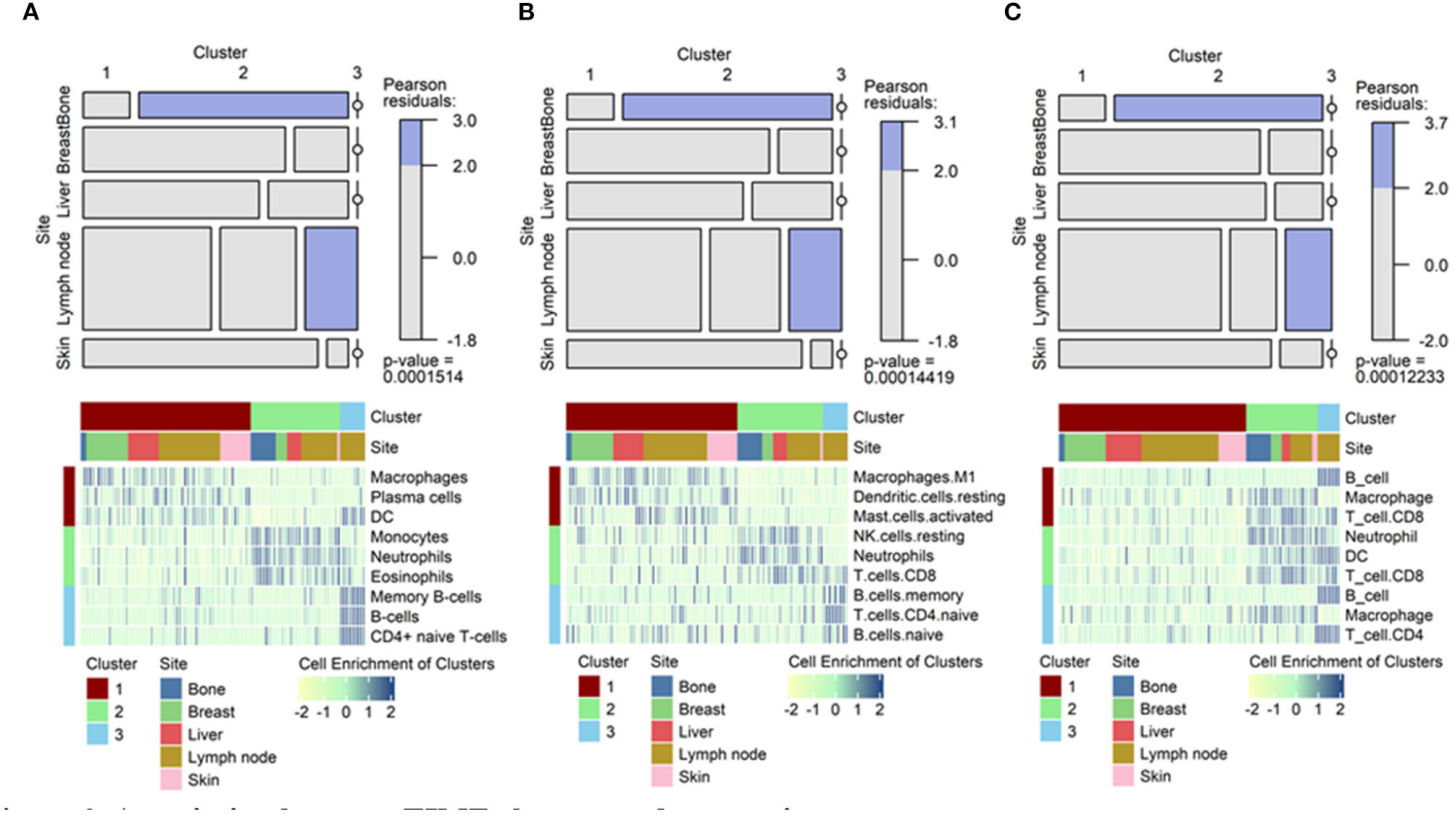
Association between TIME clusters and tumor sites. Mosaic plots showing the association of specific clusters with specific metastatic sites. Heatmaps showing top 3 immune cells enriched in each cluster. For example, macrophages showed high enrichment in cluster 1, neutrophils in cluster 2, and B-cells in cluster 3 in the xCell analysis. **(A)** Results from xCell. **(B)** Results from CIBERSORT. **(C)** Results from TIMER. TIME, tumor immune microenvironment.

Tumors were then clustered according to TIME cell types to identify any association between TIME-based clusters and metastatic sites. Three clusters were identified via hierarchical clustering of TIME cell enrichment scores. In the xCell analysis, cluster 1 was associated with enriched macrophages, plasma cells, and dendrocytes. Cluster 2 was associated with enriched monocytes, neutrophils, and eosinophils. Cluster 3 was associated with enriched B-cells and CD4+ T-cells. The clusters based on TIME were significantly associated with metastatic tumor sites, particularly bone and lymph node lesions, in all 3 analyses (Figures 3A–C; *p* < 0.001, chi-square = 30.818 for xCell; *p* < 0.001, chi-square = 30.937 for CIBERSORT; and *p* < 0.001, chi-square = 31.338 for TIMER, Supplementary Table 3). Most of metastatic bone lesions were included in cluster 2. Although metastatic lymph nodes were included in all clusters, cluster 3 contained only metastatic lymph nodes.

We subsequently examined whether this finding was recapitulated in another dataset (GSE56493). As in the previous analyses, heatmaps and volcano plots were created (Supplementary Figure 2). Metastatic lymph nodes showed significantly fewer macrophages than breast lesions in the xCell analysis (*p* = 0.034, W = 560). This was also observed in the TIMER analysis (Supplementary Figure 3A, *p* < 0.001, W = 663). Metastatic bone lesions contained significantly fewer macrophages than breast lesions in both the xCell and TIMER analyses (Supplementary Figure 3B; *p* = 0.003, W = 6 for xCell and *p* = 0.024, W = 16 for TIMER). Neutrophils were significantly enriched in bone metastases compared to breast lesions in the CIBERSORT analysis (Supplementary Figure 3C, *p* = 0.015, W = 81). The clusters based on TIME were significantly associated with lymph node lesions in all 3 analyses (Supplementary Figures 3D–F; *p* < 0.039, chi-square = 21.847 for xCell; *p* < 0.001, chi-square 27.693 for CIBERSORT; and *p* < 0.001, chi-square = 32.06 for TIMER, Supplementary Table 3).

### Immune Cell Types in Metastatic Lymph Nodes in scRNA-Seq

**Figure 4:**
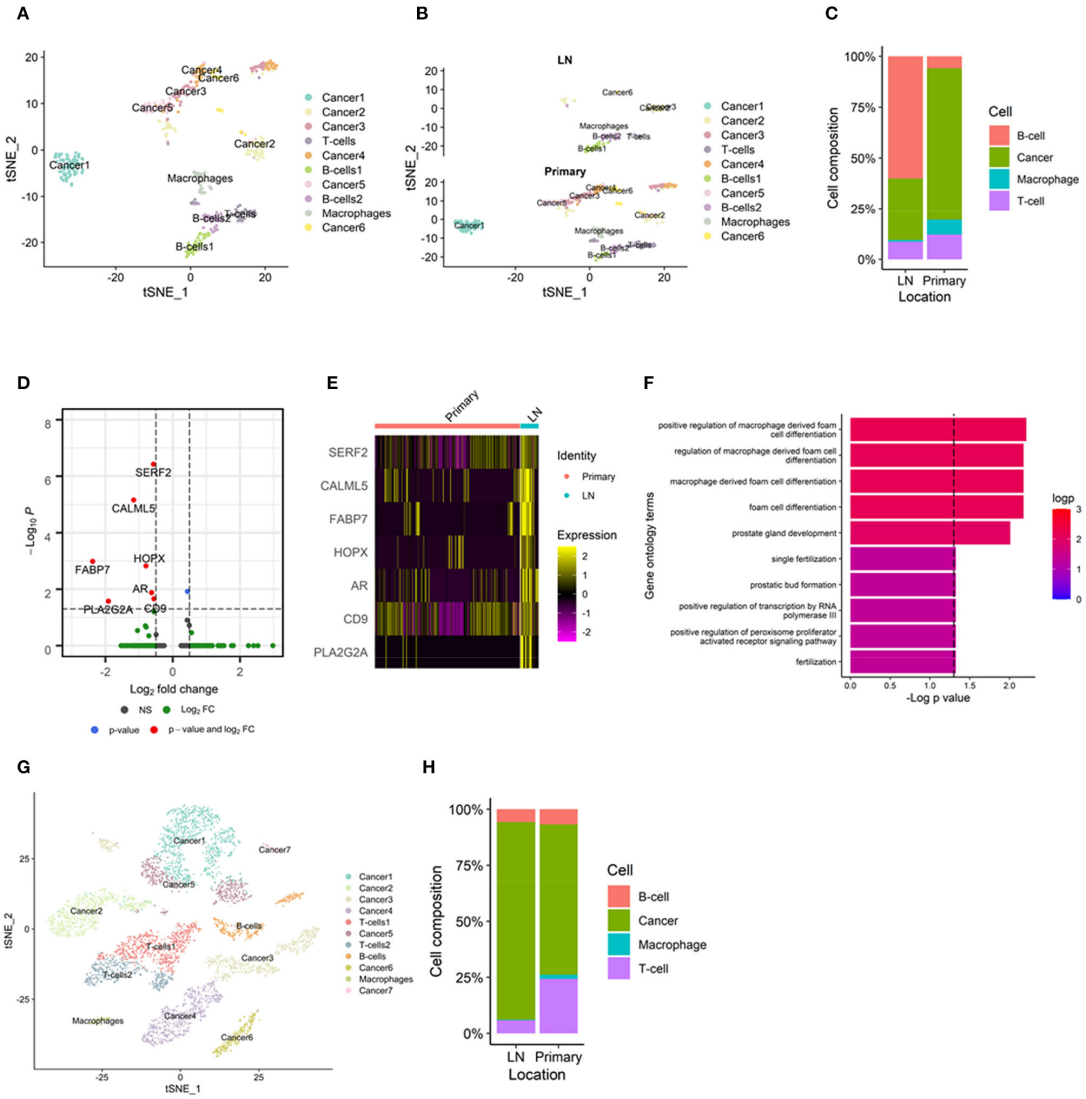
TIME of metastatic lymph nodes analyzed by single-cell RNA-sequencing. **(A)** A total of 515 cells from an scRNA-seq dataset clustered into 10 clusters and 4 cell types. **(B)** There were fewer macrophages in metastatic lymph nodes than in primary tumors in the t-SNE plot. **(C)** Bar plot showing macrophages in metastatic lymph nodes and primary tumors. **(D)** Volcano plots representing seven differentially expressed genes: SERF2, CALML5, FABP7, HOPX, AR, CD9, and PLA2G2A. All the seven DEGs showed negative log2 fold change; lower expression in primary tumors than in metastatic lymph nodes. **(E)** Heatmap demonstrating the expression of each differentially expressed gene. **(F)** In gene ontology analyses, macrophage-related terms were selected as significant. **(G)** In a head and neck cancer scRNA-seq dataset, there were fewer macrophages in metastatic lymph nodes than in primary tumors in the t-SNE plot. **(H)** Bar plot showing macrophages in metastatic lymph nodes and primary tumors. AR, androgen receptor; CALML5, calmodulin-like protein 5; FABP7, fatty acid binding protein 7; HOPX, homeodomain-only protein; PLA2G2A, phospholipase A2 group IIA; scRNA-seq, single-cell RNA sequencing; SERF2, small EDRK-rich factor 2; TIME, tumor immune microenvironment; t-SNE, t-distributed stochastic neighborhood embedding.

To elucidate the differences in cell types in TIME according to the metastatic site, we analyzed single-cell-level data of metastatic lymph nodes from breast cancer (GSE75688). A total of 515 cells from 11 tumor samples were clustered into 10 clusters and 4 cell types (Figure 4A). The markers of each cell cluster and their expression are shown in Supplementary Figure 4. The TIMEs of metastatic lymph nodes had fewer macrophages than those of primary tumors (Figures 4B,C). There were more B-cells and T-cells in metastatic lymph nodes than in primary breast tumors, which was consistent with our results derived from bulk gene expression data. In addition, we analyzed whether cancer cells in different tumor sites had different gene expression, which could be affected by different TIMEs. When cancer cells from primary and metastatic tumors were compared, seven DEGs were identified: small EDRK-rich factor 2 (SERF2), calmodulin-like protein 5 (CALML5), fatty acid binding protein 7 (FABP7), homeodomain-only protein (HOPX), androgen receptor (AR), CD9, and phospholipase A2 group IIA (PLA2G2A) (Figures 4D,E). Functional enrichment analysis using GO analysis demonstrated that macrophage-related terms were significantly enriched in this transcriptional profile (Figure 4F). There were two sets of paired primary tumors and metastatic lymph nodes from the same patient, and we performed additional analyses in these paired samples. In these samples, the TIMEs of the metastatic lymph nodes had fewer macrophages than those of the primary tumors, as observed in the total dataset (Supplementary Figures 5A,B). We identified nine DEGs in these paired samples: SERF2, CALML5, FABP7, HOPX, PLA2G2A, sperm protein associated with the nucleus on the X chromosome D (SPANXD), endoplasmic reticulum oxidoreductase 1 alpha (ERO1L), AR, and CD9 (Supplementary Figures 5C,D). These genes included seven of the DEGs identified in all primary and metastatic tumors. In GO analyses, macrophage-related terms were also selected as significantly enriched (Supplementary Figure 5E).

To validate our finding of the difference in the enrichment of macrophages in TIME, we performed additional analyses using single-cell-level transcriptome data derived from head and neck cancer (GSE103322). Even though the cancer type was different from that in the other datasets, head and neck cancer also commonly metastasizes to the lymph nodes. Therefore, it was expected to show similar results, i.e., fewer macrophages and more lymphocytes in metastatic lymph nodes than in the primary tumor. A total of 5782 cells from 22 tumors were clustered into 11 clusters and 4 cell types (Supplementary Figure 6A). The markers of each cell cluster and their expression are shown in Supplementary Figure 6B. The TIMEs of metastatic lymph nodes had fewer macrophages than those of primary tumors (Figures 4G,H). In six paired samples, the TIMEs of metastatic lymph nodes had fewer macrophages than those of primary tumors, as in the total dataset (Supplementary Figures 6C,D).

## Discussion

The present study focused on differences in TIME based on metastatic sites of breast cancer. We explored the TIMEs of different metastatic sites using bulk gene expression datasets. This was followed by single-cell analysis to support differences in the TIMEs of metastatic lymph nodes, which was revealed in the analysis of the bulk datasets. The TIME showed a high variance according to the metastatic site. Myeloid cells were significantly enriched in metastatic lymph nodes and metastatic bone lesions compared to breast lesions. This was further shown by the cluster analysis, as metastatic lymph nodes and bone lesions were classified into specific clusters. Myeloid cells, including macrophages and neutrophils, are closely associated with tumorigenesis and metastasis, although they have antitumor roles. However, their protumor roles are more common, such as promoting tissue remodeling and suppressing innate immunity to tumor cells (36–40). Additionally, they may interact with each other via cytokines such as interlukin-8 or tumor necrosis factor alpha (41, 42). Although macrophages and neutrophils cooperate and have functional similarity, our study showed that the distribution of myeloid cells can vary in different metastatic tissues. This suggests that the pre-metastatic niche and TIME formation may employ different mechanisms in different metastatic sites.

It is unclear whether macrophages have protumoral or antitumoral effects. One of the difficulties in identifying the role of macrophages in metastasis is that there are various subtypes of macrophages and myeloid cells affecting TIME. Previous studies found that the abundance or immaturity of dendritic cells in lymph nodes is related to a lower antitumor effect of T-cells (43, 44). The effects of fewer macrophages in metastatic lymph nodes on metastasis may be inferred from these previous reports. It can be hypothesized that a smaller number of TAMs causes less antigen processing/presenting and decreased T-cell responses in the lymph nodes. Conversely, another subtype of macrophages induces protumoral effects by suppressing the T-cell response via the cytotoxic T-lymphocyte-associated protein 4 and programmed death-ligand 1 (PD-L1) pathways (45). Therefore, there is controversy surrounding the reason for reduced macrophages in metastatic lymph nodes. Scholars revealed that numbers of macrophages were smaller in normal lymph nodes than in primary breast tumor (46). However, another study demonstrated that macrophages in metastatic lymph nodes were lesser in normal lymph nodes, by immunohistochemistry analysis in breast cancer patients (47). Considering these previous studies, the finding that there are fewer macrophages in metastatic lymph nodes than in primary lesions may be deemed not only a result of normal immune cell distribution in lymph nodes but also feature of disease progression. This can provide an insight into the underlying biology of metastasis. For cancer cells to survive in the metastatic site, they may change the enrichment of specific immune cell types in TIME. The decrease in macrophages may result from this phenomenon.

Results from the bulk datasets were supported by scRNA-seq data of breast cancer and head and neck cancer. In a part of samples, metastatic lymph nodes showed lesser macrophages than primary tumors. Our results imply that the immune populations in TIME may be, at least partly, dependent on the anatomical site. Our results highlight that the TIMEs of specific metastatic sites may be similar, even if the primary site differs. This is supported by the concept of the pre-metastatic niche, wherein the environment of the metastatic site is altered to proper “soil” for the tumor “seed” (48).

Interestingly, there were fewer macrophages in bone metastases than in breast lesions in our datasets. However, neutrophils were enriched in metastatic bone lesions compared to breast lesions. Therefore, antigen presentation is not well-activated in bone lesions as macrophages and dendritic cells which showed lower enrichment in the bone lesions compared with breast lesions. These types of cells were associated with antigen presentation of tumors; a key process of the tumor immunity mediated by lymphocytes (49, 50). Instead, it seems that neutrophils may be a key factor in bone metastasis. Neutrophils release cytokines, including chemokine receptor type 4 and matrix metallopeptidase 9, to foster bone invasion (5). Additionally, previous studies showed that the degree of neutrophil recruitment was associated with prognosis in patients with bone metastasis (51, 52). Although the lung is another representative metastatic site of breast cancer, characteristics of TIME in metastatic lung lesions were not shown in this study due to a lack of data. However, previous animal studies have shown that TAMs and neutrophils promote metastasis of breast cancer (53, 54). These knowledges also support that TIME may vary according to the metastatic sites.

It is notable that FABP7 and AR were selected as differentially expressed genes which were upregulated in cancer cells in metastatic lymph nodes. FABP7 is known as a molecular factor affecting brain metastasis and survival/proliferation of breast cancer (55, 56). Also, AR is known to promote proliferation and migration of breast cancer (57). As previous knowledges, the present study shows potential relevance of these genes toward promoting lymph node metastasis of breast cancer, by analyzing scRNA-seq data.

Precision oncology is based on the unique genomic or molecular characteristics of each patient and tumor (58, 59). Immuno-oncology therapy can enhance the host immunity or inhibit the protumor effect of tumor-infiltrated immune cells (19, 60). Immune checkpoint inhibitor (ICI) therapies, including PD-L1 checkpoint inhibitors, are the most commonly applied immunotherapies in the clinic. ICIs have caused unprecedented prognostic improvement in lung cancer, lymphoma, and melanoma patients (61–63). However, several recent studies have reported that the response of ICI therapy differs based on the metastatic site. It has been postulated that the different immunologic environment of each tissue may affect the response to ICIs (64–66). This would indicate that the concept of precision medicine needs to move from an individual-based approach to a lesion-based approach. In other words, it is important to investigate the TIME of each metastatic lesion or site to select an appropriate treatment option, thereby improving prognosis. The presence of TAMs in TIME might be of particular interest, as targeting TAMs is thought to affect the response to ICI therapy (45). There are experimental lines of evidence that suppressing TAMs enhances the efficacy of ICIs and inhibits cancer progression (67–69).

Our findings provide insight into the clinical translation of treatment strategies that consider differences in TIME based on the metastatic site. There have been few studies documenting the diverse microenvironments of different metastatic tissues (70). This is the first study to demonstrate differences in TIME based on the metastatic site in breast cancer. We clearly showed differences in the TIME components in metastatic lymph nodes and bone lesions compared to breast lesions. Further, this finding was validated using a single-cell dataset and data from another cancer. Our data also highlight the necessity of different therapies according to the metastatic site, even in the same individual. However, we were unable to include patients with metastatic lesions in more than one anatomical site. Nevertheless, our results can be used to select treatment options based on metastatic site.

In this study, we applied multiple analysis tools to conduct quantitative analysis of immune populations in TIME. There were a few dissimilarities between three methods. In addition, some results were not completely replicated in the same analysis methods. It is caused by different deconvolutional methods and marker genes in each tool. Also, different demographics and clinical settings of each dataset may cause partly heterogeneous results. Nonetheless, the key findings were supported by similar tendency despite a degree of statistical insignificance between the three methods. It is a limitation of this study focusing on analyses for different pre-existing datasets. Further study is warranted to corroborate these findings with experimental methods.

In conclusion, we analyzed 2 gene expression datasets and one scRNA-seq dataset to analyze the TIMEs in different metastatic breast cancer sites. The enrichment of cell types in TIME differed in different metastatic sites. Specifically, there were fewer macrophages in metastatic lymph nodes and metastatic bone lesions than in breast lesions. Our findings suggest that the TIME of each metastatic lesion must be considered when selecting the optimal immuno-oncology treatment option.

## Data Availability Statement

Publicly available datasets were analyzed in this study. This data can be found at: https://www.ncbi.nlm.nih.gov/geo/, GSE124647, GSE56493, GSE75688, and GSE103322.

## Author Contributions

HL, KN, and HC designed the study, performed analysis and interpretation, and wrote the manuscript. All authors read and approved the final manuscript.

## Conflict of Interest

The authors declare that the research was conducted in the absence of any commercial or financial relationships that could be construed as a potential conflict of interest.
