# Supplementary material for: Differences in Tumor Immune Microenvironment in Metastatic Sites of Breast Cancer
Source: Front Oncol. 2021 Mar 18;11:649004. doi: 10.3389/fonc.2021.649004 (PMC8013993; doi:10.3389/fonc.2021.649004)
Supplement: Supplementary file 1 [file Data_Sheet_1.docx]

**Supplementary Materials**

**Differences in tumor immune microenvironment in metastatic sites of breast cancer**

**Supplementary figures**

**Supplementary tables**

**Supplementary Figures**

**
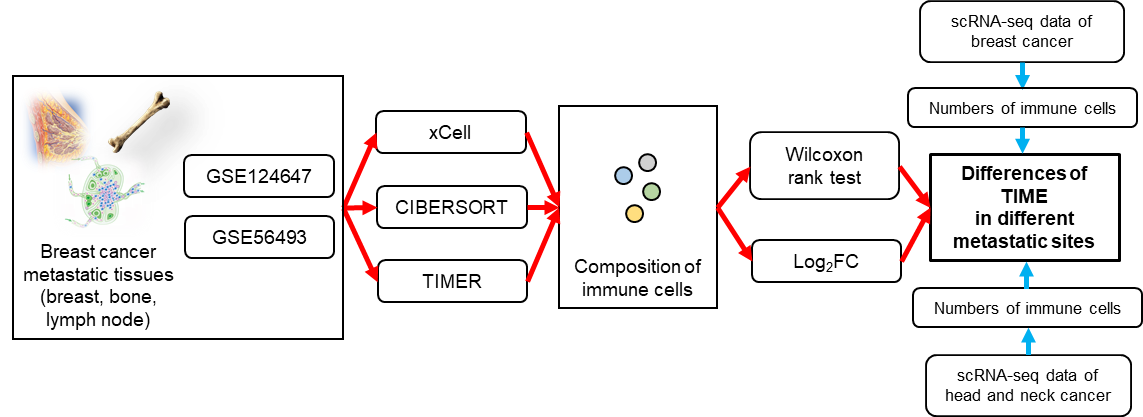
 Supplementary Figure S1. The overall study scheme.**

The overall study scheme of the present study is represented.

**
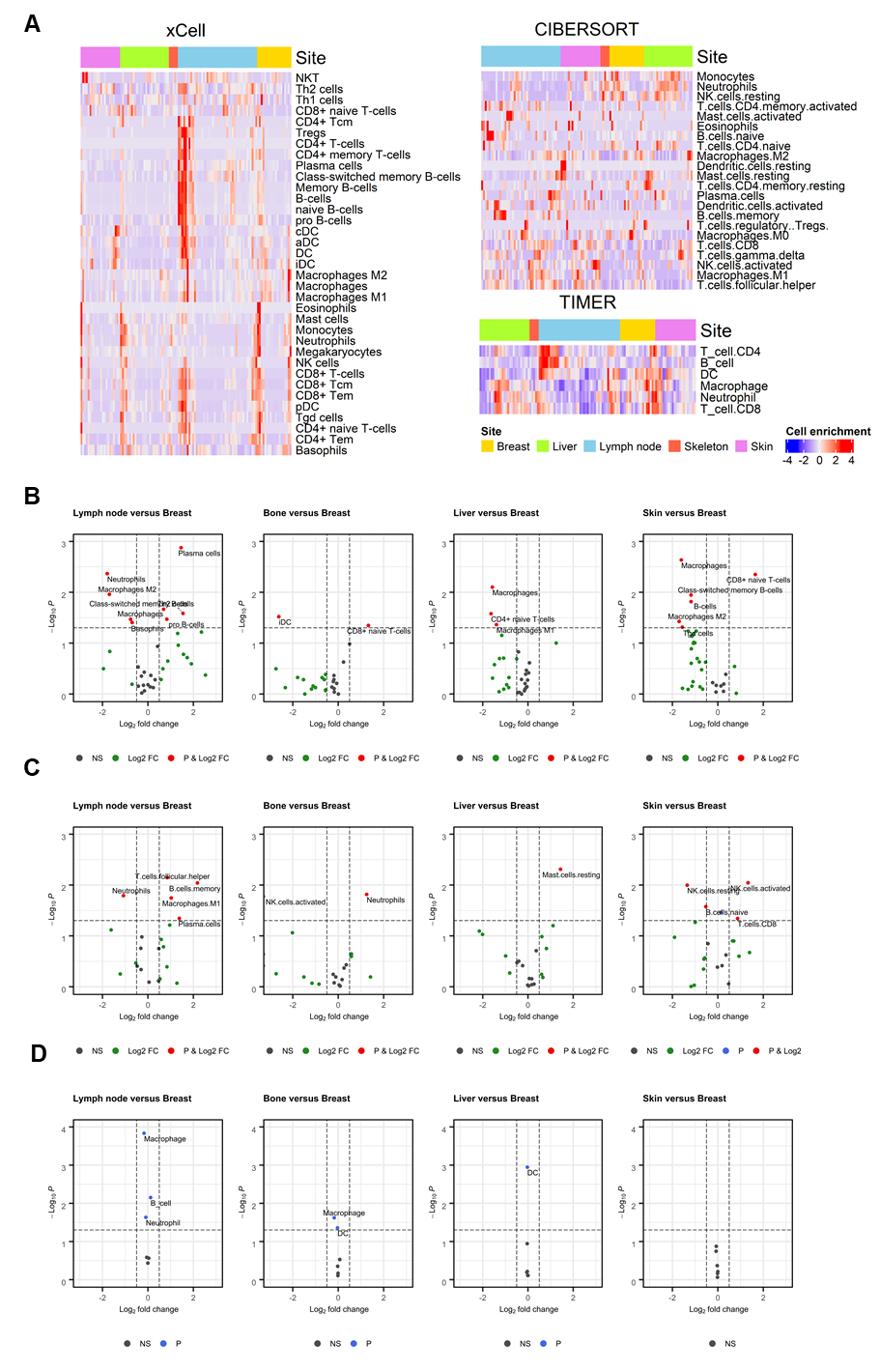
Supplementary Figure S2. Enrichment of immune cell populations in different metastatic sites in another microarray dataset (GSE56493).**

**(A)** Heatmaps depicting the distribution of immune cell enrichment scores according to the metastatic site estimated using xCell, CIBERSORT, and TIMER. The distribution of immune cells varied across all samples. **(B-D)** Volcano plots showing enriched immune cells in specific metastatic sites compared to the breast lesions. Wilcoxon rank test was applied to compare the enrichment of immune cells. A threshold of log_2_FC was 0.5 to determine differential expression, and a p-value of 0.05 was considered significant. Values outside the range of x-axis were displayed at the margin of plots. Results from the xCell, CIBERSORT, and TIMER analyses are shown in panel **B**, **C**, and **D**, respectively. Notably, there were fewer macrophages in metastatic lymph nodes than in breast lesions, and there were more neutrophils in metastatic bone lesions than in breast lesions.

**
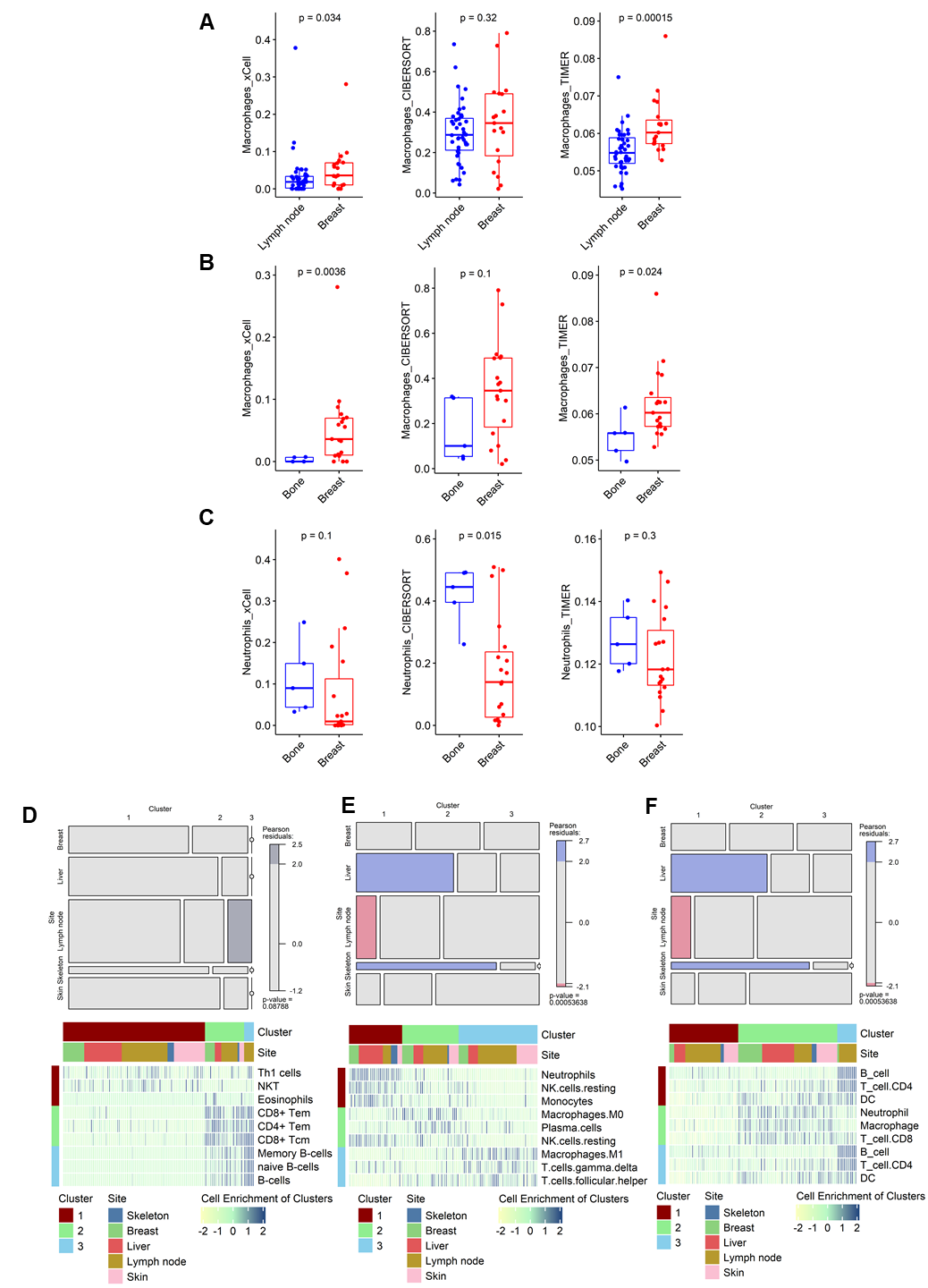


Supplementary Figure S3. Different composition of myeloid cells according to the metastatic tumor site in another microarray dataset (GSE56493).**

**(A)** In the xCell and TIMER analyses, macrophages showed significantly lower enrichment scores in metastatic lymph nodes than in breast lesions (p = 0.034, CI = 0.29*10^-5^~4.01*10^-2^ and p = 0.00015, CI = 2.92*10^-3^~9.23*10^-3^, respectively). **(B)** In the xCell and TIMER analyses, macrophages showed significantly lower enrichment scores in metastatic bone lesions than in breast lesions (p = 0.0036, CI = -0.07~-0.04*10^-1^ and p = 0.024, CI = -1.26*10^-2^~-9.36*10^-4^ , respectively). **(C)** In the CIBERSORT analysis, neutrophils showed significantly higher enrichment scores in metastatic bone lesions than in breast lesions (p = 0.015, CI = 0.08~0.42). **(D-F)** Mosaic plots showing the association of specific clusters with specific metastatic sites. Heatmaps showing top 3 immune cells enriched in each cluster. Results from the xCell, CIBERSORT, and TIMER analyses are shown in panel **D**, **E**, and **F**, respectively.

**
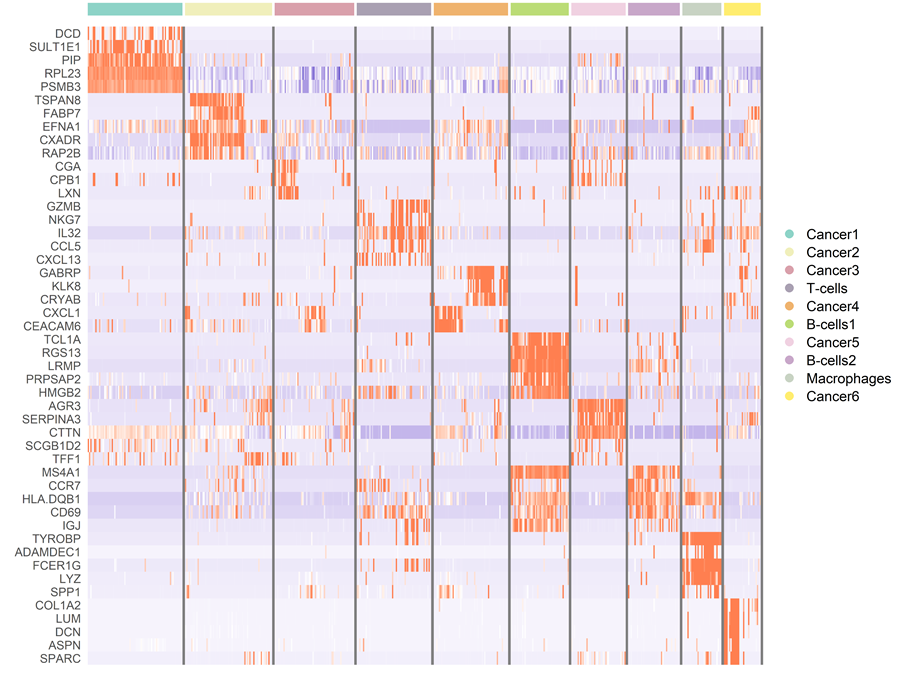
Supplementary Figure S4. The markers of each cluster and their expression in a scRNA-seq data (GSE** **GSE75688).**

The markers of each cell cluster and their expression are represented.

**
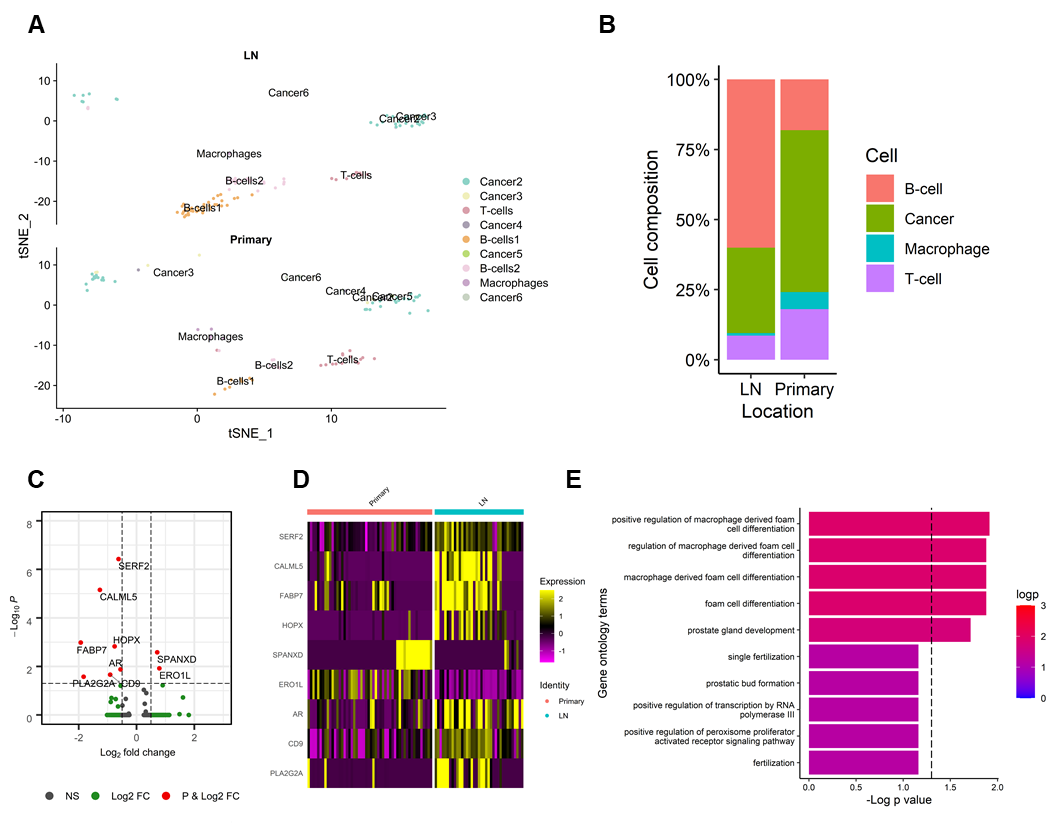
Supplementary Figure S5. TIME of metastatic lymph nodes analyzed by single-cell RNA-sequencing in paired breast cancer samples.**

**(A)** There were fewer macrophages in metastatic lymph nodes than in primary tumors in the t-SNE plot. **(B)** Bar plot showing macrophages in metastatic lymph nodes and primary tumors. **(C)** Volcano plots representing nine differentially expressed genes: SERF2, CALML5, FABP7, HOPX, PLA2G2A, SPANXD, ERO1L, AR, and CD9. **(D)** A heatmap demonstrating the expression of each differentially expressed gene. **(E)** In gene ontology analyses, macrophage-related terms were selected as significant.

AR: androgen receptor; CALML5: calmodulin-like protein 5; ERO1L: endoplasmic reticulum oxidoreductase 1 alpha; FABP7: fatty acid binding protein 7; HOPX: homeodomain-only protein; PLA2G2A: phospholipase A2 group IIA; scRNA-seq: single-cell RNA sequencing; SERF2: small EDRK-rich factor 2; SPANXD: sperm protein associated with the nucleus on the X chromosome D; TIME: tumor immune microenvironment; t-SNE: t-distributed stochastic neighborhood embedding


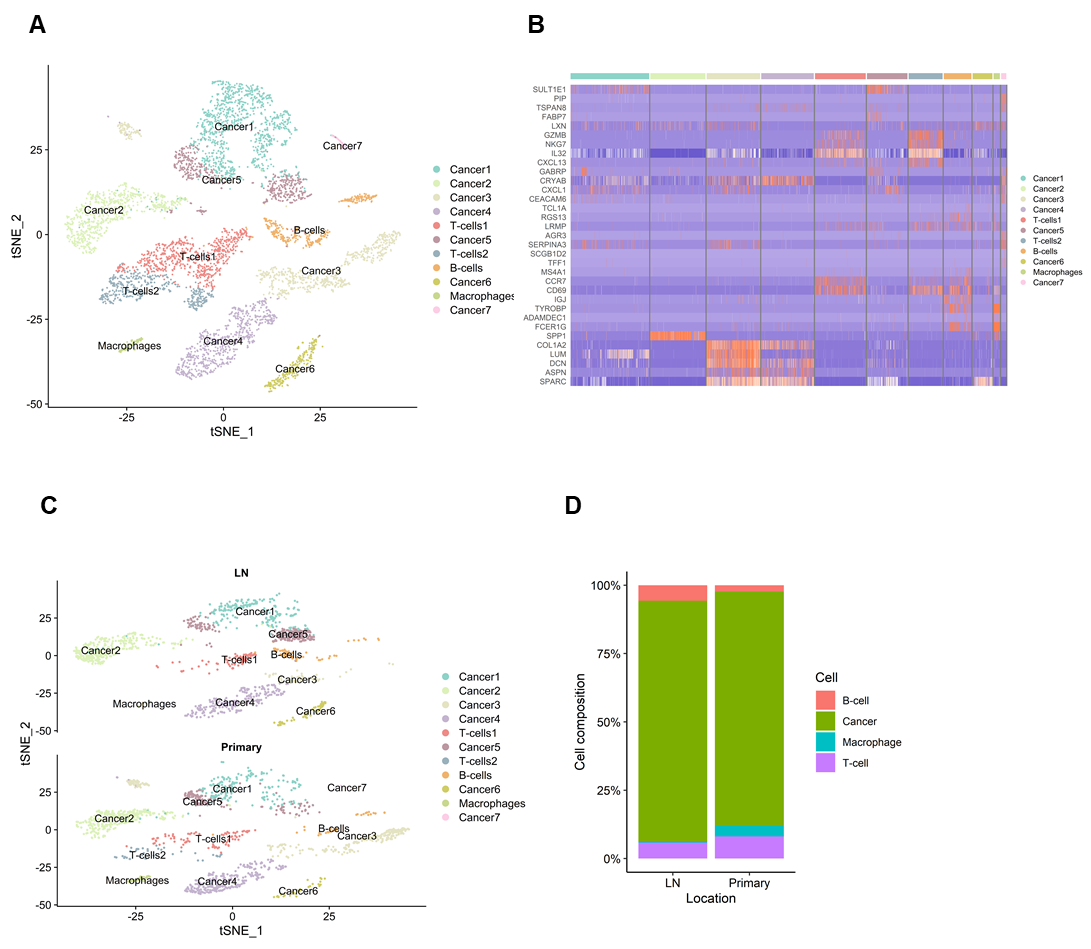
**Supplementary Figure S6. TIME of metastatic lymph nodes analyzed by single-cell RNA-sequencing in head and neck cancer samples.**

**(A)** 5782 cells from total 22 tumor samples were clustered into 11 clusters and 4 cell types. **(B)** The markers of each cell cluster and their expression were represented. **(C)** In paired head and neck cancer samples, numbers of macrophages were less in metastatic lymph nodes than in primary tumors in the t-SNE plot. **(D)** The bar plot showed fewer macrophages in metastatic lymph nodes.
t-SNE: t-distributed stochastic neighborhood embedding

**Supplementary Tables**

**Supplementary Table S1: The number and biopsy sites of samples included in this study.**

|  | **Breast** | **Lymph node** | **Bone** | **Liver** | **Skin** | **Excluded subjects** | | | | **Total** |
| --- | --- | --- | --- | --- | --- | --- | --- | --- | --- | --- |
|  |  |  |  |  |  | **Lung/pleura** | **Ovary** | **Soft tissue** | **Others** |  |
| **GSE124647** | 19 | 44 | 11 | 16 | 12 | 4 | 1 | 32 | 1 | 140 |
| **GSE56493** | 19 | 44 | 5 | 27 | 22 | 2 | 0 | 0 | 1 | 120 |

**Supplementary Table S2: Detailed statistics of comparison analyses in each dataset and method.**

A separate Excel file is provided.

**Supplementary Table S3: Cross table of cluster analysis**

| **GSE124647** | | | | |
| --- | --- | --- | --- | --- |
| **TIMER** | | **1** | **2** | **3** |
| **xCell** | **CIBERSORT** |  | | |
| **1** | **1** | 50 | 3 | 0 |
|  | **2** | 4 | 2 | 0 |
|  | **3** | 2 | 0 | 0 |
| **2** | **1** | 7 | 2 | 0 |
|  | **2** | 4 | 19 | 0 |
|  | **3** | 0 | 0 | 0 |
| **3** | **1** | 0 | 0 | 0 |
|  | **2** | 0 | 0 | 2 |
|  | **3** | 1 | 0 | 6 |

| **GSE56493** | | | | |
| --- | --- | --- | --- | --- |
| **TIMER** | | **1** | **2** | **3** |
| **xCell** | **CIBERSORT** |  | | |
| **1** | **1** | 5 | 18 | 0 |
|  | **2** | 23 | 10 | 0 |
|  | **3** | 15 | 16 | 0 |
| **2** | **1** | 0 | 10 | 0 |
|  | **2** | 0 | 2 | 0 |
|  | **3** | 0 | 6 | 6 |
| **3** | **1** | 0 | 0 | 0 |
|  | **2** | 0 | 0 | 0 |
|  | **3** | 0 | 0 | 6 |
